# An Optical Fiber-Based Nanomotion Sensor for Rapid Antibiotic and Antifungal Susceptibility Tests

**DOI:** 10.1021/acs.nanolett.3c03781

**Published:** 2024-02-05

**Authors:** Jiangtao Zhou, Changrui Liao, Mengqiang Zou, Maria Ines Villalba, Cong Xiong, Cong Zhao, Leonardo Venturelli, Dan Liu, Anne-Celine Kohler, Sergey K. Sekatskii, Giovanni Dietler, Yiping Wang, Sandor Kasas

**Affiliations:** † Laboratory of Physics of Living Matter (LPMV), École Polytechnique Fédérale de Lausanne (EPFL), CH-1015 Lausanne, Switzerland; ‡ Department of Health Sciences and Technology, ETH Zurich, 8092 Zurich, Switzerland; § Guangdong and Hong Kong Joint Research Centre for Optical Fiber Sensors and Key Laboratory of Optoelectronic Devices and Systems of the Ministry of Education and Guangdong Province, College of Physics and Optoelectronic Engineering, Shenzhen University, Shenzhen 518060, China; ∥ Laboratory of Biological Electron Microscopy (LBEM), École Polytechnique Fédérale de Lausanne (EPFL), and Department of Fundamental Biology, Faculty of Biology and Medicine, University of Lausanne (UNIL), CH-1015 Lausanne, Switzerland; ⊥ International Joint Research Group VUB-EPFL BioNanotechnology & NanoMedicine, 1050 Brussels, Belgium; ◆ Centre Universitaire Romand de Médecine Légale, UFAM, Université de Lausanne, 1015 Lausanne, Switzerland

**Keywords:** Optical fiber sensor, nanomotion device, antibiotic/antifungal susceptibility test, two-photon polymerization

## Abstract

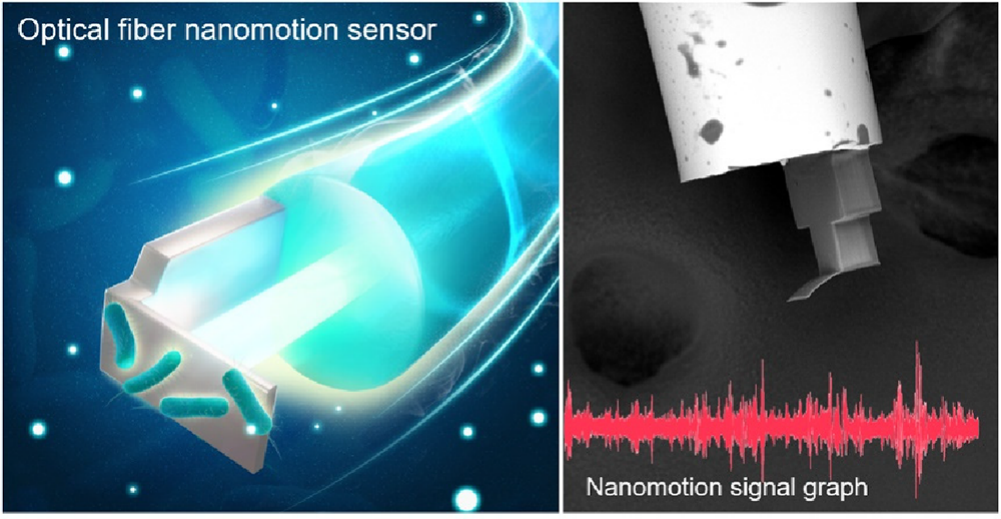

The emergence of antibiotic and antifungal resistant microorganisms represents nowadays a major public health issue that might push humanity into a post-antibiotic/antifungal era. One of the approaches to avoid such a catastrophe is to advance rapid antibiotic and antifungal susceptibility tests. In this study, we present a compact, optical fiber-based nanomotion sensor to achieve this goal by monitoring the dynamic nanoscale oscillation of a cantilever related to microorganism viability. High detection sensitivity was achieved that was attributed to the flexible two-photon polymerized cantilever with a spring constant of 0.3 N/m. This nanomotion device showed an excellent performance in the susceptibility tests of *Escherichia coli* and *Candida albicans* with a fast response in a time frame of minutes. As a proof-of-concept, with the simplicity of use and the potential of parallelization, our innovative sensor is anticipated to be an interesting candidate for future rapid antibiotic and antifungal susceptibility tests and other biomedical applications.

Proliferation of bacteria and yeasts that resist more and more antibiotics and antifungals is a worldwide public health issue.^1,2^ This issue in recent decades has induced an increase in hospitalizations, mortality rates, and costs of medical treatments. So far, many strategies have been developed to control these microbial proliferation such as the novel molecules or the more targeted use of antimicrobial drugs.^2^ However, the wide use of antimicrobial drugs has unfortunately led to widespread antimicrobial resistance among these microorganisms, and thus the spread of antimicrobial resistances has outpaced the development of new antimicrobial drugs.^3^

An important approach to limit their spread is to diagnose drug-resistant bacteria by the antimicrobial susceptibility test.^2^ Such tests drastically reduce the use of large-spectrum antibiotics that are documented to induce resistance and guide effective strategies of treatment. Yet, the main problems for many current susceptibility testing approaches are the speed of the test and the parallelization for multiple tests.^1^ Typical susceptibility tests take tens of hours for identifying drug-resistant microorganisms, and traditional sensitivity tests based on the bacterial and fungal proliferation rate require at least 24 h to complete in the case of rapidly growing microorganisms but can last up to one month in the case of bacteria such as *Mycobacterium tuberculosis* or *Bordetella pertussis*. A rapid antimicrobial susceptibility test is of importance for promoting antimicrobial stewardship and epidemiological surveillance.^4^ Such tests should permit a quick identification of the most appropriate drug to fight against a specific organism, ideally in a time frame of 1–3 h.

Several years ago, we developed an atomic force microscopy (AFM)-based nanomotion sensor in the susceptibility test to assess the viability of microorganisms upon a given drug in a time frame of minutes.^5^ This test was performed by monitoring the vibrations, referred to as nanomotion, of an AFM cantilever onto which living organisms of interest, including bacteria, yeast, plant and mammalian cells, were immobilized.^6^ The dynamic nanometer-scale vibration of cantilever attributes to multiple activities of living organisms such as the metabolism, extracellular organelles, ion channels, and other membrane motions.^7,8^ Thus, the cantilever vibration would immediately stop once the microbial viability is compromised, as a response to the antimicrobial drugs.^6^ This AFM nanomotion sensor was implemented in a number of rapid antibiotic and antifungal susceptibility tests,^8−10^ and cellular activity of single cells,^7^ including cancer cell sensitivity to chemotherapeutics,^11^ as well as life-detecting vibrations.^6^

Despite these advantages, the AFM nanomotion sensor needs to be improved due to the complex and sophisticated instruments as well as the expertise required in operation, including, for example, a precise laser alignment before each susceptibility test.^12^ This is because of the difficulty in parallelization that significantly limits large-scale tests for the diagnosis of bacteria or fungi. To dates, many efforts have been made to advance this technology, but most are in the framework of AFM devices.^12−14^ Recently, we simplified the nanomotion sensor, out of the framework of AFM, by tracing single yeast cell nanomotion that correlates to its cellular activity with an optical microscope,^7^ but this prototype compromised the sensitivity and was found to be difficult in studying bacteria-related nanomotion.

In this work, we present an optical fiber-based nanomotion sensor that shows a high sensitivity of nanomotion detection in antimicrobial susceptibility tests and indicates the potential of parallelization because of its compact feature and simplicity of use. A flexible 3D-printed cantilever as a nanomotion sensing beam was anchored at the distal end of the optical fiber by two-photon polymerization (2PP) technology. Typically, optical fiber sensors are popular in physiochemical applications due to their compactness and fast-response,^15−17^ and the recent 2PP technique provides direct writing of various microscale structures^18,19^ with features as thin as 200 nm,^20^ and their combination had led to interesting implementations such as a nanoindentation sensor^21^ and soft microrobots.^22^ In our sensor, the vibration of the cantilever onto which the microorganisms are attached is real-time monitored by an in-fiber interferometer^23^ implemented at the end of the optical fiber. The geometry and nanomechanical properties of our cantilever sensors, i.e., a thickness of about 1 μm and a spring constant of 0.3 N/m, are comparable to that of the AFM nanomotion cantilever. As a proof-of-concept prototype, this novel device shows excellent performance in real-time susceptibility tests of *Escherichia coli* and *Candida albicans* to antibiotics and antifungals, respectively. This strategy of a nanomotion sensor with both advantages in fast-response and parallelization may advance this technology toward a next-generation nanomotion sensor for large-scale and rapid antimicrobial susceptibility tests, as well as other technological and biomedical applications.

The schematic representation of our optical fiber-based nanomotion sensing system is shown in Figure 1. A laser beam at a single wavelength of 1550 nm was led into the optical fiber and then amplified by an erbium-doped fiber amplifier (EDFA). Then, the polarization of the amplified laser within fiber was modified by a polarization controller (PC), before splitting into two beams through a 3 dB coupler. One of the divided laser beams propagated until it reached the sensing end face of the optical fiber, on which the 2PP-printed flexible cantilever was anchored at a distance. Then, the incident beam induced two independent reflections: one reflected at the fiber end face and the other reflected at the 2PP-printed cantilever across the microcavity in-between (Figure 1). These two reflected laser beams were coupled into the optical fiber and then interfered as a Fabry-Pérot (FP) interferometer. Assuming a broadband incident laser was used, this would lead to an interference pattern with fringes as indicated in Figure 1 due to the difference in the optical path between two beams. Because living microorganisms induce the nanomotion vibration of the cantilever in the medium, such vibration varies the optical path difference between two reflected beams, and correspondingly leads to the dynamic shifting of the interference spectrum, whereas for the incident laser at a fixed wavelength, the microorganism-related cantilever vibration can induce variation of the output optical intensity. Finally, this dynamic variation of reflected laser intensity was recorded by a photodetector. Therefore, one can real-time assess the viability of living microorganism in the susceptibility assays by monitoring the fluctuation intensity of an output interference laser as a function of time.

**Figure 1:**
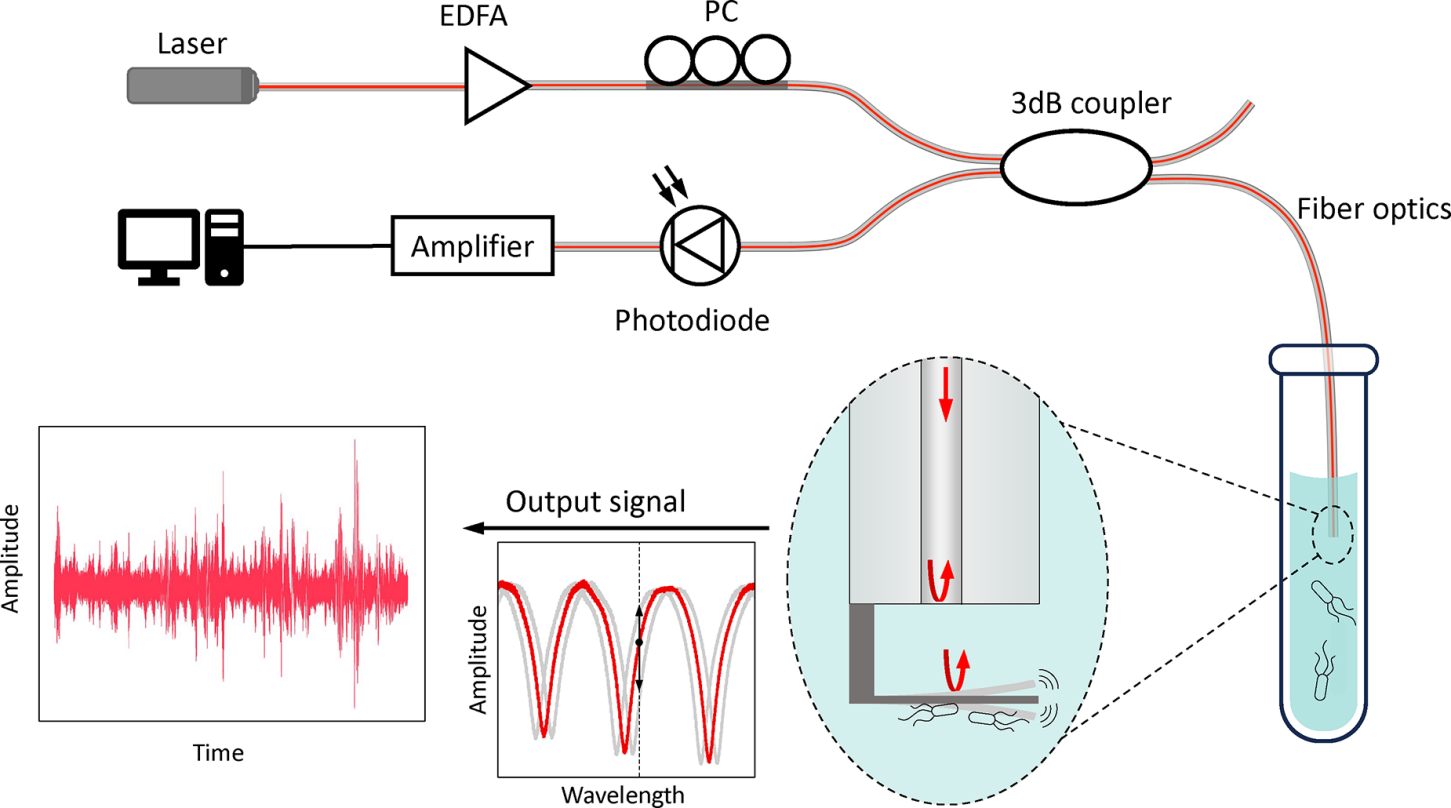
Schematic representation of our proposed optical fiber based nanomotion sensor system, and the working principle of detecting the microorganism nanomotion induced cantilever vibration as an optical interferometer.

To achieve a high detection sensitivity of cantilever vibration, we realized several enhancements in our system: first, we applied the polarization controller to match the laser wavelength at the sharp edge of the spectrum (Figure 1, inset), and therefore, a tiny cantilever vibration would be maximized on the optical intensity variation. Further, we optimized the cantilever fabrication and obtained a relatively balanced power of two reflected laser beams that results in a high contrast on the interference spectrum. Moreover, we compressed the free spectral range (FSR) of the interference pattern by enabling a larger length of microcavity. The FSR is the spacing in optical wavelength between two successive optical intensity maxima or minima on the spectrum, which relates to the optical path difference between two reflected beams:
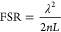
1where λ is the light wavelength, *L* is the length of microcavity, and *n* is refractive index of the medium in the cavity. We note that a longer microcavity could lead to a smaller FSR. These enhancements in the system together contribute to a more sensitive and precise detection of cantilever vibration in the acoustic hood (Figure S1).

## The Fabrication of Optical Fiber-Based Nanomotion Sensors

**Figure 2:**
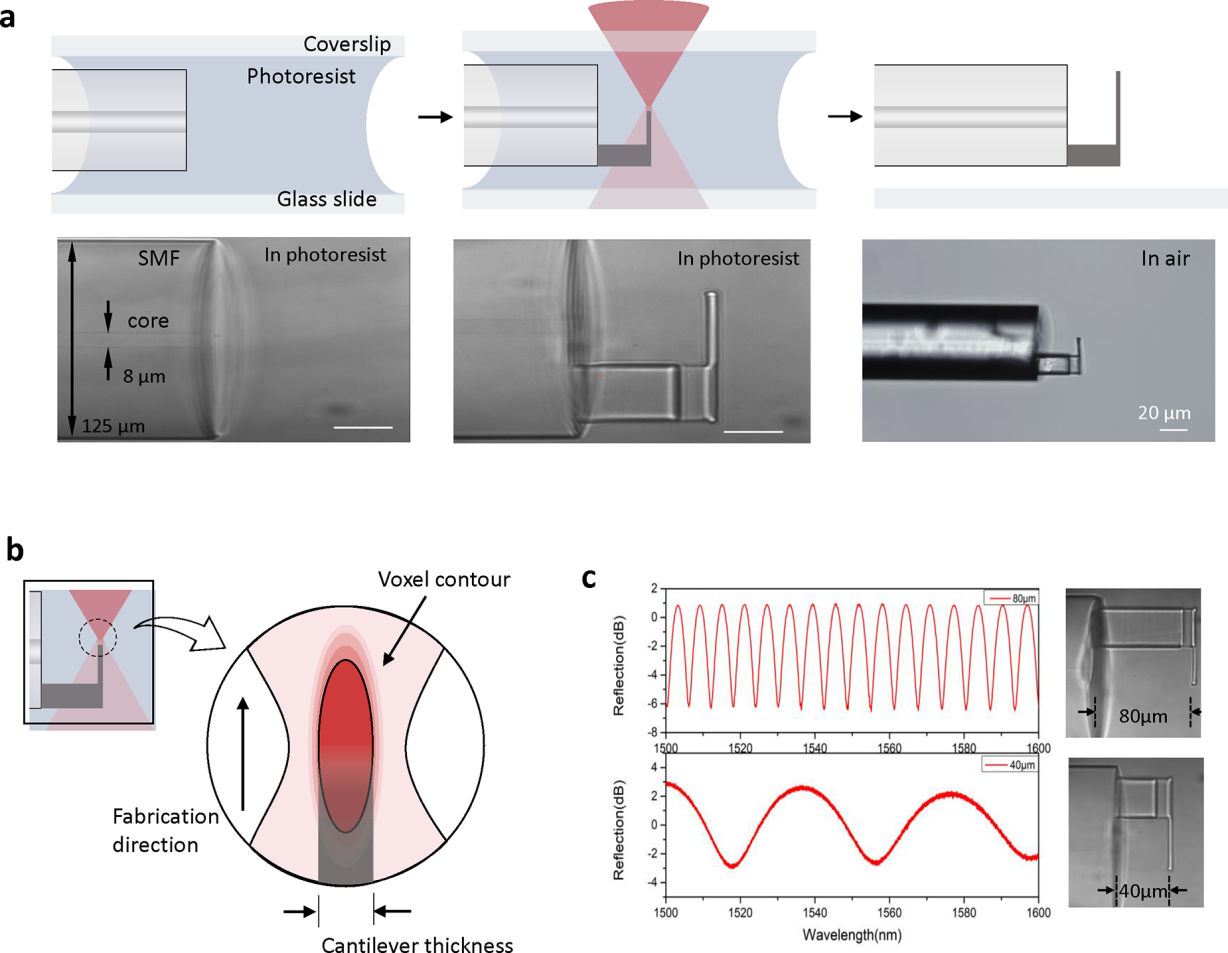
Fabrication of a optical fiber-based nanomotion sensor. (a) The schemes and optical images during the cantilever fabrication process under the two-photon polymerization system. Scale bars are 20 μm. (b) Schematic of the cantilever fabrication. (c) The optical spectra of the nanomotion sensor with different lengths of microcavity.

The fabrication of our optical fiber nanomotion sensor head is demonstrated in Figure 2a. First, a cleaved commercial single-mode optical fiber (SMF), with a diameter of 125 μm and a core diameter of approximately 8 μm, was immersed in the negative photoresist, sandwiched between a glass slide and a coverslip. Then, this setup was mounted onto an air-bearing stage for the following two-photon polymerization process, in which a femtosecond (fs) laser with a pulse width of 290 fs and a pulse repetition rate of 200 kHz was performed. A relatively low power femtosecond laser (2 mw) was applied during the polymerization process. The fabrication of the cantilever started from the base layer-by-layer in the direction of the fiber axis, and the height of this base corresponds to the length of the microcavity. At the top of the base, a rectangular cantilever was then polymerized. Lastly, the polymerized structure on the fiber tip was immersed in a mixture solution with acetone and isopropyl alcohol to remove the residual photoresist, and thus we obtained the 2PP printed nanomotion sensor.

Remarkably, to achieve a high-sensitivity cantilever, we have optimized several parameters in this 2PP technique during the cantilever fabrication. First, the sensitivity of the nanomotion sensing cantilever relates to its spring constant:
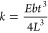
2where *E* is the Young’s modulus of the polymerized structure, and *b*, *L*, and *t* are the width, length, and thickness of this rectangular cantilever, respectively. Due to the size of the optical fiber, significantly optimizing the geometry of the cantilever such as the width and length is limited. Therefore, we implemented 2PP-printing in the vertical direction to achieve a thinner cantilever. As seen in Figure 2b, the minor axes of elliptical voxel contour that is around 200–400 nm is perpendicular to the fabrication direction of the cantilever, referring to the single layer thickness of cantilever, and this printing strategy enables better control of the cantilever thickness. However, we found that the single layer cantilever was difficult to implement in practice due to the high surface tension in microcavity during the washing and drying processes that induce cantilever distortion, and therefore two- or three-layer printed cantilevers were fabricated with a thickness of around 1 μm. Besides, the relatively low power of the femtosecond laser and the relatively fast scanning velocity in the polymerization process enable a lower intrinsic stiffness of a polymerized structure that relates to its Young’s modulus and therefore enlarged the spring constant and the detection sensitivity of the cantilever.

Further, the fabricated nanomotion sensors were characterized by collecting their interference spectra with a broadband light source. As seen in Figure 2c, the spectra showed uniform interference fringes but different FSR for different length of microcavity. From eq 1, the height of this base in this 2PP-printed structure, that is, the length of the microcavity in the FP interferometer, corresponds to the FSR in the spectrum. A smaller FSR would improve the detection sensitivity of cantilever deflection, and therefore a relatively large base height, ca. 80 μm, was applied in this nanomotion sensor.

**Figure 3:**
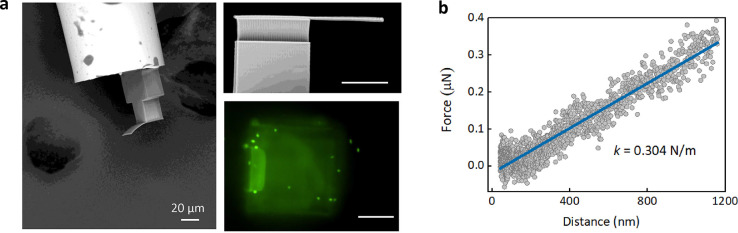
SEM images and mechanical property characterization of the 2PP-printed cantilever. (a) SEM images of the sensing head of our optical fiber based nanomotion sensor and the fluorescence image of the living *E. coli* stained on the cantilever. Scale bars are 20 μm. (b) The mechanical measurement on the 2PP-printed cantilever showed a spring constant of 0.304 N/m.

Scanning electron microscopy (SEM) images of our optical fiber-based nanomotion sensor are shown in Figures 3a and S2. As seen, a thin rectangular cantilever is anchored at the end-face of the optical fiber, and this flexible cantilever is perpendicular to the fiber end-face with a microcavity in-between. Remarkably, by using the direct nanoindentation measurement technique, the mechanical property of this cantilever is characterized, showing a spring constant as low as approximately 0.3 N/m (Figure 3b). This value is in the same order of magnitude of the commercial silicon nitride AFM cantilever. We believe this high flexibility of our cantilever attributes to the optimized geometry and fabrication process, such as the thickness of cantilever and the Young’s modulus of polymerized material that is in the order of hundreds MPa or several GPa, lower than that of commercial AFM cantilever (hundreds of GPa).

## Detection of Bacterial Resistance to Antibiotics

As a proof of principle, we applied our proposed nanomotion device to the susceptibility study of two species of microorganisms: one species of bacteria, *Escherichia coli* (*E. coli*, Gram-negative, motile bacteria) and another species from the fungi kingdom, *Candida albicans* (*C. albicans*, pathogenic yeast). By exposing the microorganisms to specific antimicrobials, we monitored the output laser intensity of optical fiber to trace the dynamic cantilever fluctuation, and therefore to access the real-time viability of microorganisms upon drug exposure, as in our previous studies.^5,6,9^ To perform the antimicrobial susceptibility tests, we carried out nanomotion sensing in a custom acoustic hood to minimize vibrational noise, and a z-stage motor was applied to ensure a minor disturbance on the cantilever while gently immersing into the medium (Figure S1).

**Figure 4:**
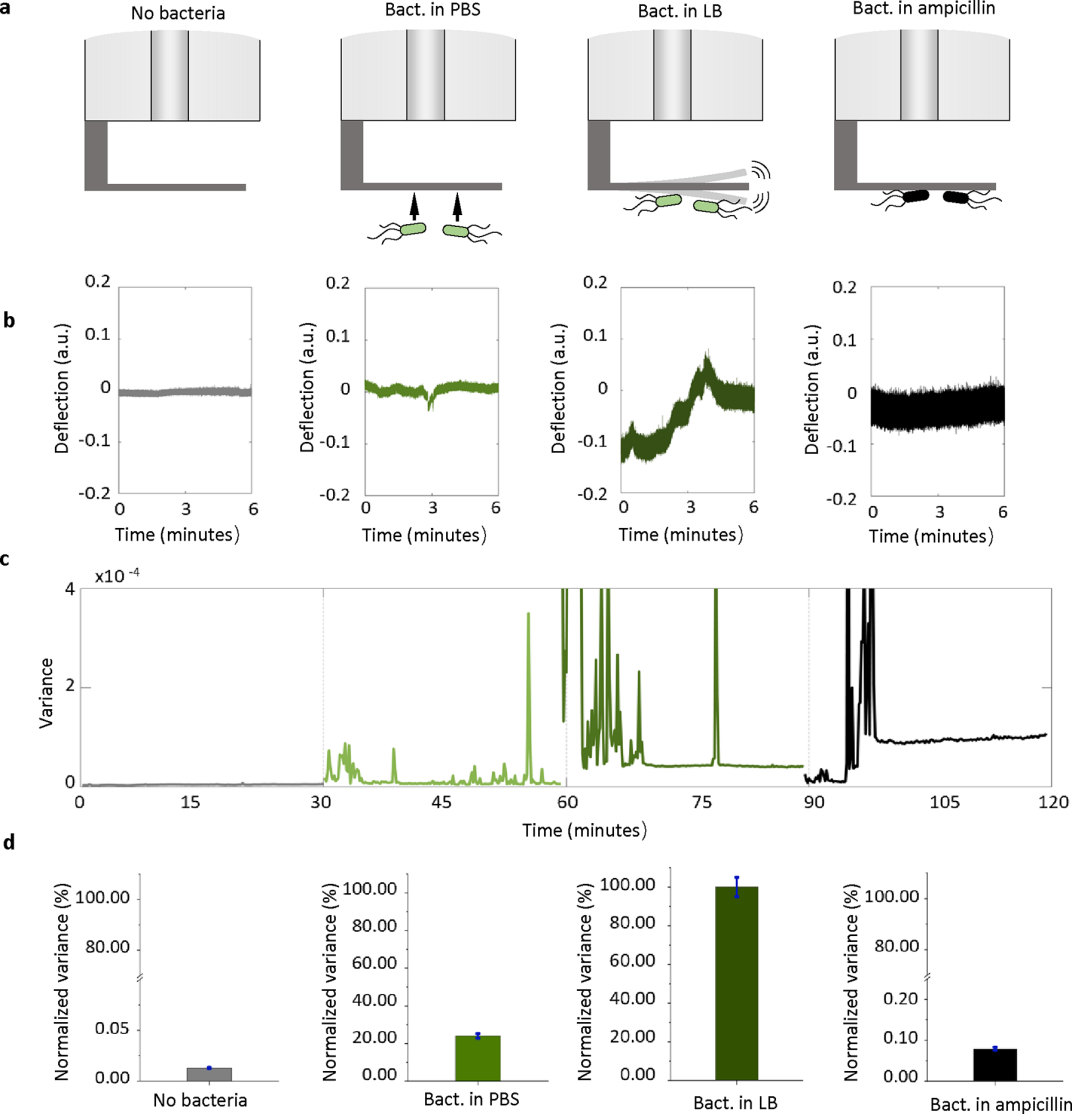
Nanomotion detection assay of *E. coli* susceptible to ampicillin by using the optical fiber nanomotion sensor. (a) Schematic representation of the workflow of this nanomotion detection: bare 2PP-printed cantilever in PBS media; bare cantilever in LB solution; the cantilever attached with *E. coli* in the LB solution; and the *E. coli* attached cantilever in LB solution with the addition of ampicillin. (b) Representative deflection signals of the cantilever in culture medium (gray), during the attachment procedure (red), and of the cantilever with attached *E. coli* before (green) and after drug exposure (black). (c) Variance of the deflection filtered signals. The signal variance was calculated using data from 10-s-long segments and plotted as a function of time. (d) Normalized variance averages after processing for different experimental conditions. The green bar represents the 100% value, determined from the variance values calculated before the exposure to the drug and the black bar the variance average calculated after the exposure to antifungal, which induced a significant reduction in the fluctuations.

In the first assay, we investigated a strain of *E. coli* (DH5α) that is susceptible to ampicillin. As shown in Figure 4, the nanomotion detection assay was performed as the following workflow: the first step was to obtain the dynamic fluctuation of bare cantilever in phosphate buffered saline (PBS) buffer; then, *E. coli* was attached to the cantilever and traced in the PBS buffer; afterward, this dynamic fluctuation was monitored in the substituted lysogeny broth (LB) nourishing medium; and finally this monitoring was continued in the LB solution containing 16 μg/mL ampicillin.

Figure 4b,c shows the results of the representative cantilever dynamic vibration and the variance evolution during the workflow. As seen, the bare cantilever showed a low dynamic vibration in PBS buffer. This low vibration may be attributed to sub-nanometric surrounding noise in liquid as this fluctuation immediately enlarged after liquid-to-air transferring (Figure S3). The corresponding variance of this noise-induced cantilever fluctuation remained minimal. The signal of vibration and its variance can be immediately detectable after bacteria is attached on the cantilever, exhibiting a strong fluctuation of cantilever deflection as well as time-dependent variance. Afterward, these vibrational signals and the variance became significantly enlarged after transferring into the substituted LB medium. This indicates the sustained viability of this microorganism throughout the experiment. However, upon the injection of the LB medium containing ampicillin, the fluctuation of cantilever quickly slowed down, and the variance showed a decrease due to their gradual loss of biological activities as a function of time, until unnoticeable variation 15 min later that indicates the death of bacteria. This observation agrees with the previous experiments^9^ indicating that ampicillin produced reduction of fluctuations 20 min after drug injection. This result is confirmed by the normalized variance in Figure 4d.

It is noticeable that the amplitude of cantilever vibration after ampicillin injection is larger than that of a bare cantilever (Figure 4c). We believe this is due to the variation of refractive index in the medium that was induced by the chemicals released from dead bacteria, and therefore it slightly shifted the spectra and increased the baseline of output vibration signal, according to eq 1. Nevertheless, this phenomenon would not affect the diagnosis in the susceptibility test, as the normalized variance (Figure 4d) showed a significant difference between different phases. We also showed the fluorescence and SEM images (Figures 3a and S4) to prove the presence of dead and living *E. coli* binding onto the printed cantilever.

### Detection of Yeast Cellular Activity

Another trial of our optical fiber nanomotion sensor consisted of investigating the cellular activities of *C. albicans* and their response to antifungals. Similar to our previous study,^24^ we first collected the vibration of bare cantilever in the yeast-extracted peptone-dextrose (YPD) medium. Afterward, we attached the *C. albicans* cells onto the functionalized cantilever and then monitored the cantilever fluctuation in the YPD medium. Eventually, we killed the yeast cells using the antifungal caspofungin (CASP) and continued the data acquisition of cantilever vibration to monitor *C. albicans* viability in this antifungal.

**Figure 5:**
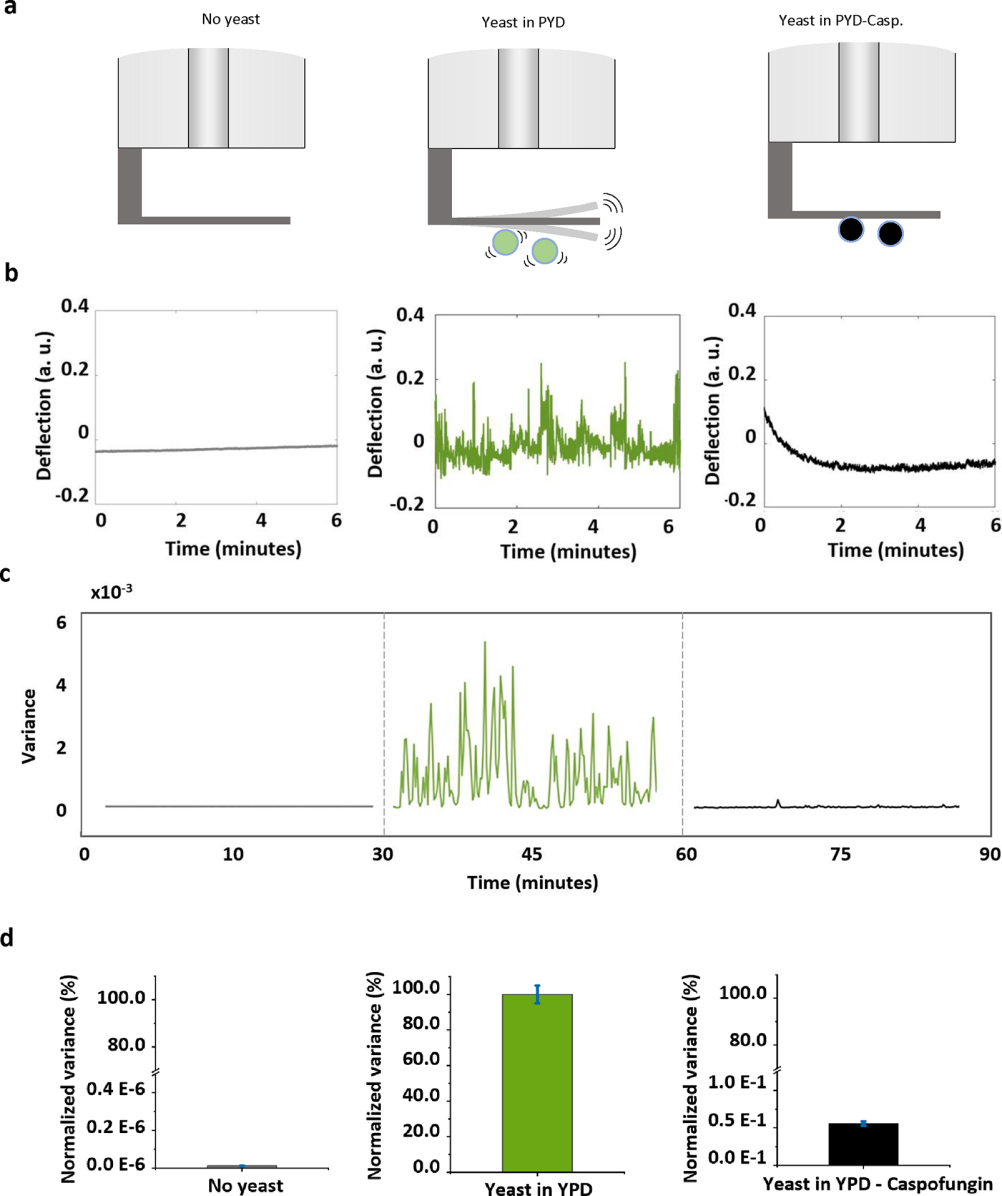
Susceptibility assay of *C. albicans* cells to antifungal caspofungin by using our optical fiber nanomotion sensor. (a) Schematic representation of the detection workflow: bare cantilever in YPD media; the cantilever attached with *C. albicans* in the YPD solution; and the cantilever with *C. albicans* attached in YPD medium with 100 μg/mL caspofungin. (b) Representative deflection signals of the cantilever in culture medium (gray) and cantilever with attached yeast before (green) and after drug exposure (black). (c) Variance of the processing deflection signals. The signal variance was calculated using data from 10-s-long segments and plotted as a function of time before (green) and after (black) yeast attachment exposure to the drug. (d) Normalized variance averages for different experimental conditions. The green bar represents the 100% value, determined from the variance values calculated before the exposure to the drug, and the black bar the variance average of the exposure to antifungal, which induced a significant reduction in the fluctuations.

The time-dependent nanomotion signals throughout the assay, including the cantilever deflection and variance, are shown in Figure 5. In the initiating phase, the bare cantilever in liquid showed a small fluctuation and a low variance, whereas, after *C. albicans* attached to the cantilever, the vibration signal dramatically soared as well as the variance (Figure 5b–d). This huge difference in variance contract is due to the stronger cellular activity of yeast^7,24^ compared to bacteria. Lastly, once the yeast cells were killed by the caspofungin, the nanomotion signal and its variance significantly dropped and remained minimal, indicating the dysfunction and the loss of the viability of *C. albicans* cells. These variance and the normalized variance plots (Figure 5c,d) significantly point to the signal of living yeast in the YPD growth medium.

In summary, we presented a high-sensitivity, optical fiber-based nanomotion sensor for fast antimicrobial susceptibility tests with simplicity of use and the possibility of parallelization. To the best of our knowledge, it is the first optical fiber nanomechanical sensor in the assay of detecting the viability of living microorganisms and their susceptibility to antimicrobials. As a proof of principle, we proved that our optical fiber-based nanomotion sensor is capable of detecting nanometer or subnanometer cantilever vibration induced by the metabolic activities of microorganisms including typical bacteria and fungi, such as *E. coli* and *C. albicans*. We also detected the life-death transition in the susceptibility assay of these microorganisms upon exposure to antibiotics and antifungals, respectively.

An important advantage of our nanomotion sensor is the rapid antimicrobial susceptibility tests in the time frame of minutes. As AFM nanomotion sensors,^12,14^ this feature greatly speeds up the diagnosis of drug-resistant microorganisms, much faster than traditional susceptibility tests. This mainly is attributed to the excellent mechanical properties^5,25^ of 2PP-printed cantilever (*k* = 0.3 N/m), and meanwhile this sensitivity also allows the monitoring viability of small microorganisms such as bacteria. Another advantage of our nanomotion sensor is the high feasibility of parallelization for large-scale applications due to its simplicity and compactness. Besides, instead of the precise laser alignment of each test in the AFM nanomotion sensor, our in-fiber interferometric detecting method ensures that our nanomotion sensor is easy-to-use and requires no complex devices nor expertise in operation. Moreover, such an optical fiber sensing head with 2PP fabricated cantilever can be easily replaced at a low cost in practice.

In the future, a compelling yet challenging endeavor of our optical fiber nanomotion sensor is to explore the presence of various bacterial species on the cantilever. This requires sophisticated experimental designs including an accurate number of cells from different species on the cantilever as well as complex signal interpretation algorithms. It is noticeable that nanomotion signals depend on multiple factors such as the quantity and species of bacteria, the strength of their attachment, and their metabolic state. Artificial intelligence-based algorithms have the potential to decode such signals, though this capability would require extensive preliminary training based on numerous experiments to permit the algorithm to accurately assess the influence of various parameters contributing to the nanomotion signal.

Our development of optical fiber nanomotion sensor provides an easy-to-use and parallelizable approach for the fast and high-sensitivity susceptibility test that may pave the way for the implementation in hospitals for antibiotics and antifungal susceptibility assays. We anticipate our device could be an interesting candidate for next-generation nanomotion sensors for antimicrobial susceptibility tests, microorganism viability assays, and other biological and biomedical applications.

## Supporting Information Available

Additional information on the materials and methods for the experimental procedures, the optical fiber nanomotion device system, as well as the additional data of the sensor probe and bacterial attachment with the sensor (PDF)
