# Supplementary material for: An Optical Fiber-Based Nanomotion Sensor for Rapid Antibiotic and Antifungal Susceptibility Tests
Source: Nano Lett. 2024 Feb 5;24(10):2980–8. doi: 10.1021/acs.nanolett.3c03781 (PMC10941246; doi:10.1021/acs.nanolett.3c03781)
Supplement: Supplementary file 1 — nl3c03781_si_001.pdf [file nl3c03781_si_001.pdf]

Supporting information for

An optical fiber-based nanomotion sensor for rapid antibiotic and antifungal susceptibility tests

Jiangtao Zhou[1,2,†,*], Changrui Liao[3,†,*], Mengqiang Zou[3], Maria Ines Villalba[4], Cong Xiong[3], Cong Zhao[3], Leonardo Venturelli[1], Dan Liu[3], Anne-Celine Kohler[1], Sergey K. Sekatskii[1,4], Giovanni Dietler[1], Yiping Wang[3,*], Sandor Kasas[4,5,6,*]

1. Laboratory of Physics of Living Matter (LPMV), École Polytechnique Fédérale de Lausanne (EPFL), CH-1015 Lausanne, Switzerland
2. Department of Health Sciences and Technology, ETH Zurich, 8092 Zurich, Switzerland
3. Guangdong and Hong Kong Joint Research Centre for Optical Fiber Sensors and Key Laboratory of Optoelectronic Devices and Systems of the Ministry of Education and Guangdong Province, College of Physics and Optoelectronic Engineering, Shenzhen University, Shenzhen 518060, China
4. Laboratory of Biological Electron Microscopy (LBEM), École Polytechnique Fédérale de Lausanne (EPFL) and University of Lausanne (UNIL), CH-1015 Lausanne, Switzerland
5. International Joint Research Group VUB-EPFL BioNanotechnology & NanoMedicine, 1050 Brussels, Belgium
6. Centre Universitaire Romand de Médecine Légale, UFAM, Université de Lausanne, 1015 Lausanne, Switzerland

† These authors contributed equally

* Corresponding authors: jiangtao.zhou@hest.ethz.ch, cliao@szu.edu.cn, ypwang@szu.edu.cn, sandor.kasas@epfl.ch

Material and method:

Materials and reagents:

For *E. coli* susceptibility assay, all chemicals including PBS buffer, LB medium, glutaraldehyde, and the antibiotics ampicillin were supplied by Sigma-Aldrich (USA). The susceptible strains of *E. coli* in this study was ATCC 25922. For *C. albicans* viability assay, the yeast-extracted peptone-dextrose (YPD) medium is from Fisher Scientific (USA), other chemicals including glutaraldehyde and (3-Aminopropyl)triethoxysilane (APTES) are from Sigma-Aldrich (USA). The *C. albicans* isolate 101 and CEC 3675 were kindly provided by Salomé Leibundgut and Christophe D'Enfert laboratories.

2PP fabrication of cantilever fabrication:

The process of printing the optical fiber-based nanomotion sensor by femtosecond laser 2PP was divided into three steps. First, the end face of a standard single mode fiber (SMF-28, Corning Inc.) was cut flat with a fiber-optic cutter and glued horizontally to a glass slide. A drop of photoresist consisting of photoinitiator (IGR-369) and monomer (SR454, SR444 and SR368) was dropped on the end face of the standard SMF and a coverslip was placed onto the SMF upper surface to stop the photoresist from flowing away. Then, the glass slide described above was then mounted on the femtosecond laser 3D air-bearing stage for structure printing. The femtosecond laser was focused in the photoresist on the fiber end face through an oil-immersion objective (Zeiss, 63x, NA=1.4). The power of the femtosecond laser (a center wavelength of 1026 nm, a pulse width of 290 fs, a pulse repetition rate of 200 kHz) measured in front of the objective was 2 mW. The femtosecond laser printing process starts inside the fiber to enhance the adhesion between the printed structure and the fiber end face. The base and cantilever structures were printed on the SMF end face according to the pre-designed scan path. The slice spacing during laser scanning was chosen to be 100 or 200 nm according to the different parts, and the scanning speed was 0.9 mm/s. Finally, a drop of the developer made by mixing isopropanol and acetone (the volume ratio is 4:1) was applied to the fiber end face to remove the unexposed photoresist. The process can be repeated if desired and after this step the designed structure is successfully printed on the fiber end face.

Nanomechanical property characterization of cantilever:

To obtain the nanomechanical properties of the cantilever, an indentation test was performed on the cantilever using a nanoindentation instrument (Hysitron TriboIndenter TI980, USA). The flat punch tip was used for indentation, and the nanoindentation instrument with indenter tip was carefully calibrated before the experiment. The nanoindentation instrument is in a constant temperature and humidity environment to reduce the error generated in the test process. The optical fiber-based nanomotion sensor was fixed on the sample table of the nanoindentation instrument, and the flat punch tip gradually presses the end of the cantilever. The maximum loading force is set to 0.5 μN, the loading time is 15 s, and the unloading time is 2 s. The cantilever deflects under the action of the flat punch tip, and the force-distance

curve is recorded by the nanoindentation instrument, and then the spring constant of the cantilever was obtained by linear fitting of the force-distance curve.

Optical fiber-nanomotion detection system:

To perform the nanomotion assay, we employed a homemade nanomotion sensing system. The incident laser was provided by a laser source (Thorlabs, USA) at 1550 nm with a fiber coupler outlet, and then amplified by an Erbium-doped Fiber Amplifier (Keopsys, France). The amplified laser was coupled into the polarization controller (Thorlabs, USA) and then led into the optical coupler (Thorlabs, USA) before entering sensing head for nanomotion assay. The nanomotion assay was performed in a homemade acoustic hood for minimizing environmental noise. The optical fiber sensing head was hold on an 3D stage with the electronic controller in the Z-stage, in order to maintain a gentle dipping of the sensing head into the liquid in the Eppendorf. The reflected laser was first split by the coupler into a photodiode (Thorlabs, USA) to access the light intensity that was enlarged by a low-noise amplifier (Stanford research system, USA). The final output signal is monitored and recorded by the data acquisition system and LabVIEW program (National Instrument, USA) at a frequency of 10 kHz.

All the components in the system are placed on a Styrofoam for reducing external vibration. Each nanomotion sensor used in the nanomotion assay was characterized to ensure a good interfering spectrum, and the PC was tuned before each the assay to maximize the intensity of cantilever vibration, in order to ensure a reliable determination of the nanometric or sub-nanometric deflection of cantilever.

E. coli **susceptibility and** ***C. albicans*** **Viability assay**

The 2PP-printed cantilever on the top of optical fiber was preliminarily functionalized with 0.5% glutaraldehyde or 1% APTES for 5 min, by gently contacting with a droplet vertically. Then, the sensing head was rinsed by an aliquot (1 mL) of milli-Q water, and dried in the ambient condition for 4 min. The sensing head was then dipped into the suspension of the target microorganism in the Eppendorf for 30 min, followed by a few round dipping in and out of the culture medium. In the nanomotion assay in each conditions, the optical fiber head was immersed in the same deep from the surface by the motor to avoid the potential artifacts. The monitoring of cantilever vibration with microorganism in the medium was recorded after 5 min of stabilization of the entire system. The dynamic fluctuation of cantilever in different conditions was monitored for around 30 min at an acquisition rate of 10-20 kHz. The signal acquisition was performed by a self-developed program based on LabVIEW.

Signal processing and analysis

Deflection data were exported and processed to extract the cellular activity signal. The processing data steps were done using Signal Analyzer app of Matlab R2021a. The first applied processing step was a low-pass filter to remove high frequency data, the used parameters were: passband frequency of 0.5 $x\pi$ radians/sample, steepness of 0.85 and a stopband attenuation of 60 dB. Then, a subtraction of offsets

and trend from the signals were done by Detrend preprocessing, following a linear method. After those preprocessing steps, the deflection of the cantilevers over time were plotted. The variance over time were calculated over 10 seconds windows, this step shows the signal variation and can be used to characterize cellular viability. An additional variance value of the whole signal was calculated under the different experimental conditions, and error bar in the figures represents the mean variance of the fluctuations of cantilever with and without attached cells under different conditions (ie, without and with antibiotics) and the 5% error bar. In this analysis the variance of the cantilever with living cells attached was considered as 100% and the other values were calculated taking it as reference.

Fluorescence assay:

To confirm the viability of the *E. coli* attached on the surface of the cantilever, the sensing head was then dipped into life/dead stain solution (live/dead BacLight Bacterial Viability Kit, L7012, Thermo Fisher Scientific) for 15 min in the dark. The live/dead stain contained two different fluorescent dyes, which stains live cells green while dead cells appeared red. After the incubation, the fluorescence imaging was then completed using inverted research microscope ECLIPSE Ti2-U (Nikon, Japan) under 100X objective.

Figure S1 The optical fiber-based nanomotion sensing system

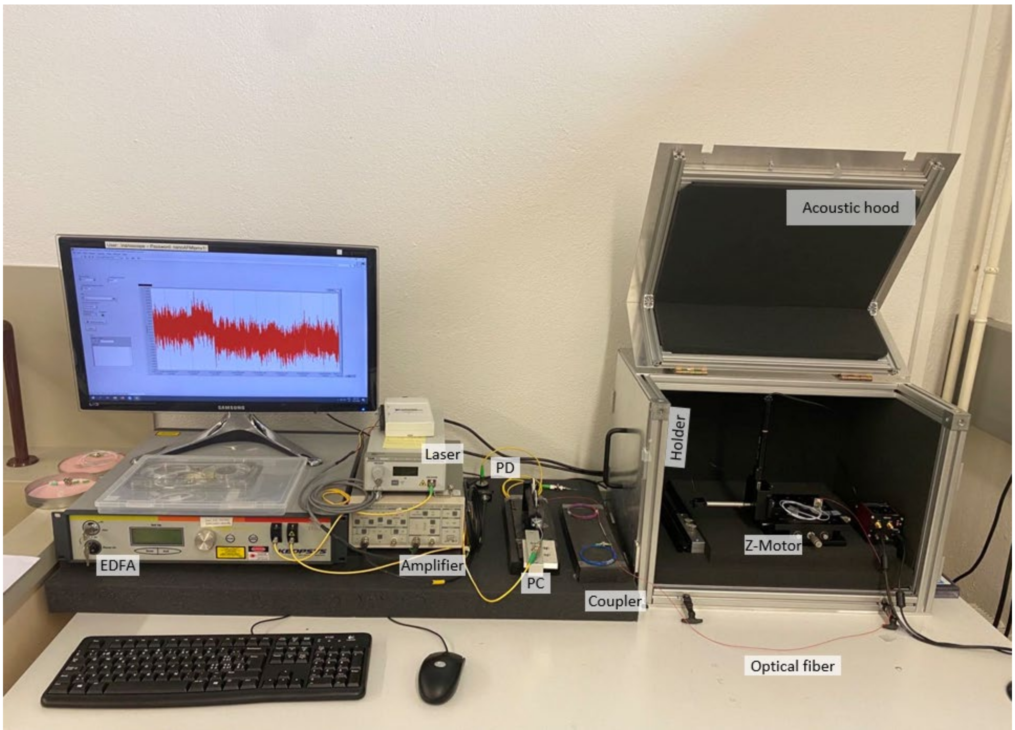

Figure S1. The homemade optical fiber-based nanomotion sensing system. All the components in the system are placed on a Styrofoam for reducing external vibration. Each nanomotion sensor used in the nanomotion assay was characterized to ensure a good interfering spectrum, and the PC was tuned before each the assay to maximize the intensity of cantilever vibration, in order to ensure a reliable determination of the nanometric or sub-nanometric deflection of cantilever.

Figure S2 Images of optical fiber-based nanomotion sensor

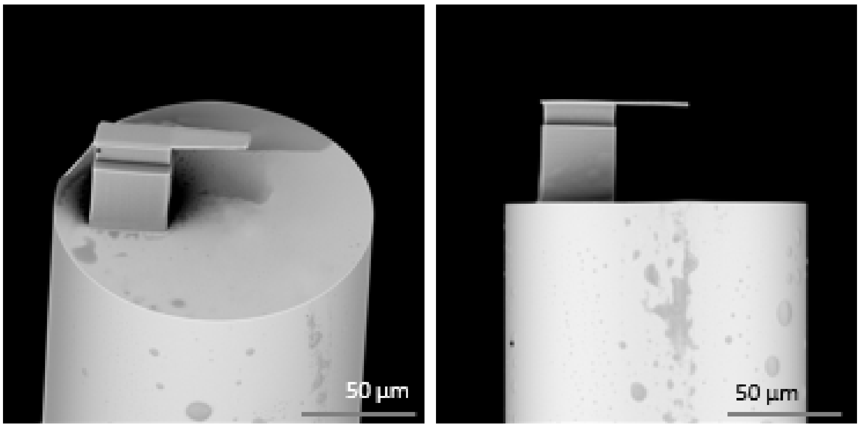

Figure S2. The SEM images of the optical fiber-based nanomotion sensor head. The different views of the optical fiber send face on which a thin and flexible cantilever was 2PP-printed and anchored with a base.

Figure S3 The optical fiber nanomotion sensor in liquid-to-air transition

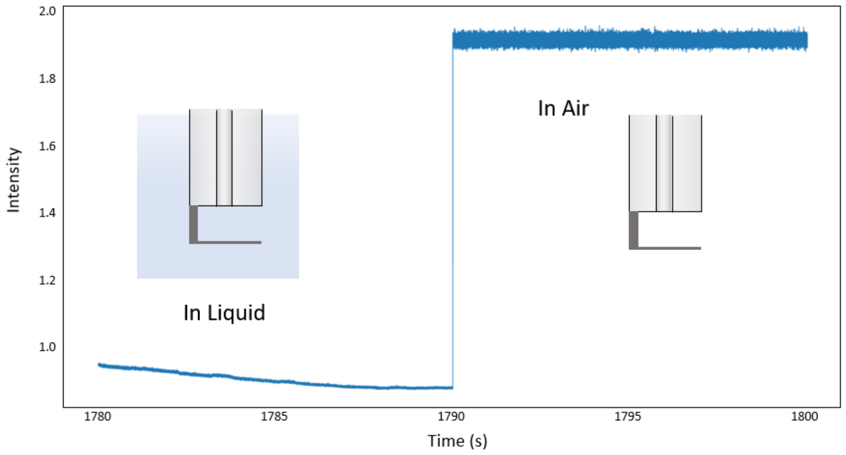

Figure S3. The signal of our optical fiber nanomotion sensor with the bare cantilever in liquid, and the transition of this signal in the transition into air by lifting the cantilever-mounted nanomotion sensor out of liquid solution. It is noticeable that the signal intensity is higher in air over that in liquid. This is because the refractive index of liquid (1.33) is closer to that of glass (1.52), compared to the air (1.00), and thus a higher ratio of light was reflected into the optical fiber while operating in air. Another interesting point is the noise of this sensor is much lower in liquid than in air, this is because the noise including the thermal and vibration noise in liquid is much lower in liquid, which benefit our following in-liquid antibiotics susceptibility study.

Figure S4. The binding between bacterial and 2PP-printed structure

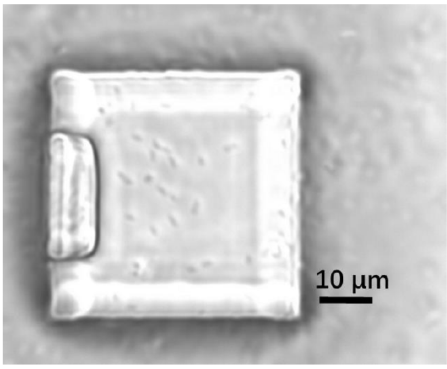

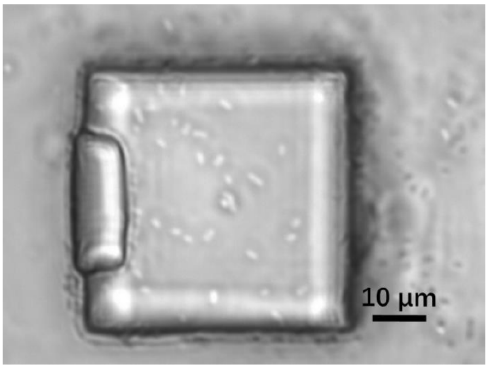

Figure S4. The SEM image of the glutaraldehyde factionalized 2PP-printed micro-structure, on the surface of which, we can see the E. coli. that captured on this 2PP-printed structure.
